# Selective delivery of 2-hydroxy APA to *Trypanosoma brucei* using the melamine motif

**DOI:** 10.1016/j.bmcl.2010.06.070

**Published:** 2010-08-01

**Authors:** Nina Klee, Pui Ee Wong, Beatriz Baragaña, Farah El Mazouni, Margaret A. Phillips, Michael P. Barrett, Ian H. Gilbert

**Affiliations:** a Division of Biological Chemistry and Drug Discovery, College of Life Science, University of Dundee, Sir James Black Centre, Dundee DD1 5EH, UK; b Division of Infection and Immunity and Wellcome Trust Centre for Molecular Parasitology, Glasgow Biomedical Research Centre, University of Glasgow G12 8TA, UK; c UT Southwestern Medical Center at Dallas 6001 Forest Park, Dallas, TX 75390-9041, USA

**Keywords:** *Trypanosoma brucei*, P2 transporter, Ornithine decarboxylase

## Abstract

*Trypanosoma brucei*, the parasite that causes human African trypanosomiasis, is auxotrophic for purines and has specialist nucleoside transporters to import these metabolites. In particular, the P2 aminopurine transporter can also selectively accumulate melamine derivatives. In this Letter, we report the coupling of the melamine moiety to 2-hydroxy APA, a potent ornithine decarboxylase inhibitor, with the aim of selectively delivering this compound to the parasite. The best compound described here shows an increased in vitro trypanocidal activity compared with the parent.

## Graphical abstract

**Figure:**
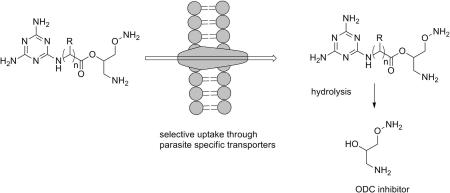

Human African trypanosomiasis (HAT), caused by two subspecies of the protozoa parasite *Trypanosoma brucei*, is an endemic disease in sub-Saharan Africa, where 60 million people are at risk. There is an urgent need for safer and more effective new drugs as current treatments of the infection show toxicity, poor clinical efficacy and an increase in drug resistance.^1^ *T. brucei* cannot synthesise purines and imports them through different nucleoside and nucleobase transporters.^2,3^ In particular, the P2 amino purine transporter is an unusual adenosine/adenine transporter unique to *T. brucei* that has a substrate recognition profile that enables it to carry molecules containing other motifs including the melamine and benzamidine moieties (Scheme 1).^4–7^ Thus, melarsoprol, diminazene and pentamidine are currently used trypanocides that are substrates of the P2 and other trypanosome transporters leading to a selectively high concentration of these trypanocidal drugs into the parasite.^7–10^

Ornithine decarboxylase (ODC) catalyzes a key step in the biosynthesis of polyamines, the conversion of l-ornithine to putrescine.^11^ Polyamines are required for cell proliferation^12^ and polyamine metabolism has been exploited successfully for the development of antiparasitic drugs. Thus, α-difluoromethylornithine (DFMO), an irreversible ODC inhibitor,^13^ is an approved drug for the treatment of African trypanosomiasis. 3-(Aminooxy)propanamine (APA), an isosteric analogue of putrescine, is another potent inhibitor of ODC and has been reported to be effective in blocking the proliferation of various tumour cells and parasites.^14–16^ A similar degree of enzyme inhibition can be expected for *T. brucei* ODC due to the highly conserved active site between the mammalian and the trypanosome ODC. In spite of this similarity in structure, the selectivity of ODC inhibitors like DFMO arises from differences in turnover rate between host and parasite enzyme and the limited capacity of *T. brucei* to scavenge the low levels of polyamines from serum.^17^ Surprisingly APA has not yet been tested for its ability to inhibit the trypanosome’s ODC, nor for its trypanocidal activity.

We have an interest in selectively delivering drugs to the trypanosome’s interior by exploiting specific transporters in the parasites plasma membrane. Several transporter systems, which recognise melamine and benzamidine motifs, have been exploited to selectively deliver toxic agents to *T. brucei*. Previously, we have reported a series of structural analogues of polyamines that coupled to melamine showed potent activity against *T. brucei* and good selectivity on a cellular level.^18,19^ More recently, we have expanded the range of trypanocidal moieties delivered to the parasite by coupling a P2 motif to nitrofurans,^20–22^ DFMO, fluoroquinolones and artesunate.^23^ In particular, the use of nitrofurans led to the identification of some very potent compounds that were curative of the trypanosome infections in mice.^20–22^

Here, we have selected 2-hydroxy APA, a close analogue of APA, for coupling to a P2 recognition unit, melamine. Both enantiomers of 2-hydroxy APA show similar enzyme-inhibition effects against mammalian ODC and the 2-hydroxy group is apparently not involved in any specific interaction.^14^ Therefore, we designed compounds where the melamine motif was attached to the 2-hydroxyl group of 2-hydroxy APA through an ester. The ester bond should be liable to hydrolysis by esterases thus releasing 2-hydroxy APA inside the parasites.

First, we synthesised APA (3-(aminooxy)propanamine)^24^ to determine its activity against the *T. brucei* target enzyme and as reference point for the in vitro activity of the corresponding melamine–APA conjugates. APA (3-(aminooxy)propanamine) was prepared following a new and shorter synthetic route. Starting from commercially available *tert*-butyl *N*-(3-bromopropyl)carbamate **1** and *tert*-butyl *N*-hydroxycarbamate in the presence of sodium hydride, BOC protected APA **2** was obtained in 42% yield. BOC deprotection was carried out with HCl in ether to afford APA hydrochloride **3** in 82% yield (Scheme 2).

2-Hydroxy APA^14^ was also prepared following a new synthetic route. Commercially available *N*-allylcarbamate **4** was oxidised with mCPBA to give the corresponding epoxide **5**.^25^ Selective opening of this epoxide using *tert*-butyl *N*-hydroxycarbamate in the presence of triethylamine in ethanol resulted in the formation of BOC protected 2-hydroxy APA **6**. As before, BOC deprotection was carried out with 2 M HCl in ether to afford 2-hydroxy APA hydrochloride **7** in 80% yield (Scheme 2).

We synthesised four melamine derivatives carrying an acid group (**9a**–**d**) by SN_Ar_ reactions. Thus, derivatives **9a**, **9b** and **9d** were obtained in good yields by reaction of glycine, β-alanine and γ-amino butanoic acid, respectively, with 2-chloro-4,6-aminotriazine **8** at 80 °C for 2 days. Displacement of the chloro group of **8** by the more hindered D,L-alanine required microwave treatment at 150 °C for 4 h to obtain the melamine derivative **9c** in 95% yield. Coupling of the melamine derivatives **9a**–**d** with BOC protected 2-hydroxy APA **6** gave rise to the 2-hydroxy APA–melamine conjugates **10a-d**. This coupling step was performed via the acid chlorides obtained by treatment of acids **9a**–**d** with thionylchloride in DCM followed by esterification with **6** in the presence of base in acetonitrile at 50 °C. Unfortunately this step was low yielding and significant amounts of the starting material **6** were recovered. As **10a**–**d** contained the BOC-protected form of 2-hydroxy APA, their BOC deprotection was carried out with HCl in ether, to afford the unprotected 2-hydroxy APA–melamine conjugates **11a**–**d** as hydrochloride salts (Scheme 3). These were kept as their hydrochloride salts, to prevent intramolecular cyclisation by attack of the amine on the carbonyl of the ester.

Firstly, the APA **3** and 2-hydroxy APA **7** were tested for inhibition of *T. brucei* ODC using a previously described assay^26^ showing that both are effective inhibitors of the enzyme with IC_50_ of 22 and 48 nM, respectively. As expected due to the highly conserved active site of ODC across species, the activities are in line with previously reported inhibition of rat liver ODC (35 and 39 nM, respectively).^14^

Compounds were tested against two *T. brucei* lines in vitro. The *T. brucei brucei* wild type that is proficient in the P2 transporter and a TbAT1 knockout line lacking the P2 transporter. This TbAT1 knockout line has been described previously.^27^ The results are summarised in the Table 1.

APA and 2-hydroxy APA displayed poor activity against the wild type strain of *T. brucei* (EC_50_ >100 μM). This is probably due to the poor uptake of these doubly charged diamines. We therefore investigated whether coupling to a melamine motif might increase activity through selective delivery via the P2 transporter. Various analogues with a melamine motif were investigated including different chain lengths separating the P2 motif (melamine) and APA. In addition, one example (**11c**) with a methyl group in the linker which may slow the rate of hydrolysis was included. The analogues (with the exceptions of **11a** and **11b**) showed a moderate increase in potency against *T. brucei*. This could be suggestive of uptake through the P2 or related transporters followed by hydrolysis in the trypanosome by esterases to release 2-hydroxy APA. In addition, compounds were evaluated against the P2 deficient strain *TbAT1* KO. Compounds **11c** and **11d** showed a reduction in activity against this cell line, further suggesting that the P2 transporter played some role in their uptake. However these compounds all show relatively weak activity.

Our previous experience with the P2 transporter has indicated that where a compound has inherent activity against the trypanosome, attaching a P2 motif can significantly increase the activity (e.g., the nitroheterocycles),^20–22^ whilst in cases where the compound does not have inherent potency against the parasite, attaching the P2 motif does not have a large effect (e.g., fluoroquinolones).^23^ APA derivatives might be expected to have activity due to their potent inhibition of ODC, although a key difference between APA and DFMO relates to the fact that APA is a competitive reversible inhibitor of the enzyme while DFMO causes irreversible inactivation after covalently alkylating the enzyme’s active site. In this case, accumulating levels of ornithine would reach an equilibrium where APA or derivatives would be displaced from the active site and putrescine production would continue, albeit at a changed equilibrium. Other possible explanations for the small increase in trypanocidal activity upon addition of a melamine uptake motif include a failure of esterases to bioconvert the melamine derivative, degradation of the compound, for example through intramolecular attack of one of the amines onto the ester, or inability to use the P2 transporter—possibly because of the high positive charge of these compounds at physiological pH.

Compound **10b** showed weak activity in both the wild-type and knockout strains; however in this compound the amines are blocked with BOC groups removing the charge, so uptake could occur through passive diffusion and not necessarily require transporters. Compound **11c** showed the most potent activity. This could be connected to it being more hindered.

ODC is a proven drug target to treat African trypanosomiasis and APA and its close analogue 2-hydroxy APA are two potent inhibitors of the *T. brucei* ODC. 2-Hydroxy APA has been coupled to a known P2 recognition motif, melamine, through four different linkers units. While APA is inactive in vitro against *T. brucei* w.t., the 2-hydroxy APA–melamine conjugates show increases in activity of up to three-fold. These data suggest that these compounds may have enhanced uptake potential compared to the parent. The data also indicates that uptake of **11c** and **11d** may have some clear dependency on the P2 transporter since activity is reduced in the P2 knockout lines. Some compounds are highly dependent on uptake through the P2 transporter, for example berenil. However, it has been shown that there are other transporters, for example HAPT1 and LAPT1 that can also take up and concentrate compounds with a melamine or benzamidine motif.^8^ In spite of the fact that we have shown that loss of P2 can be readily achieved without apparent detriment to parasite physiology, it does not necessarily lead to resistance to melamine and benzamidine containing compounds. Hence, whilst it is important to consider the risk of resistance developing to drugs carrying such a motif, it is possible to test for this.

## Figures and Tables

**Scheme 1:**
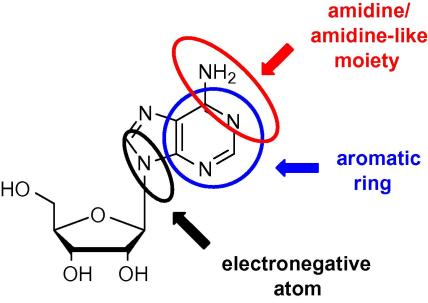
Requirements for uptake through the P2 transporter.

**Scheme 2:**
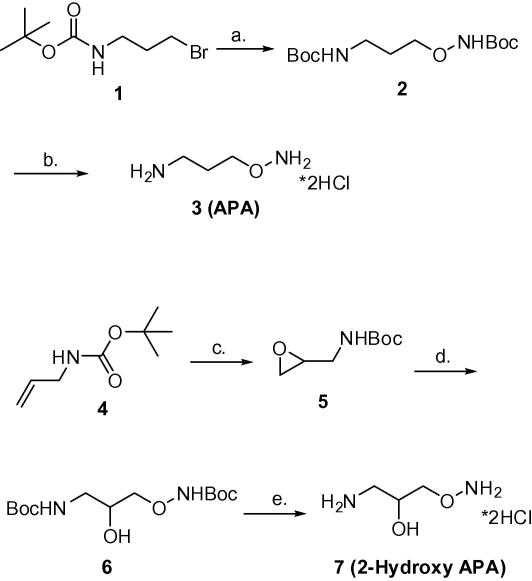
Reagents and conditions: (a) *t*BuCO_2_NHOH, NaH, THF, 0 °C, 42%; (b) 2 M HCl in Et_2_O, 82%; (c) mCPBA, DCM, 99%; (d) *t*BuCO_2_NHOH, Et_3_N, EtOH, 80 °C, 39%; (e) 2 M HCl in Et_2_O, 80%.

**Scheme 3:**
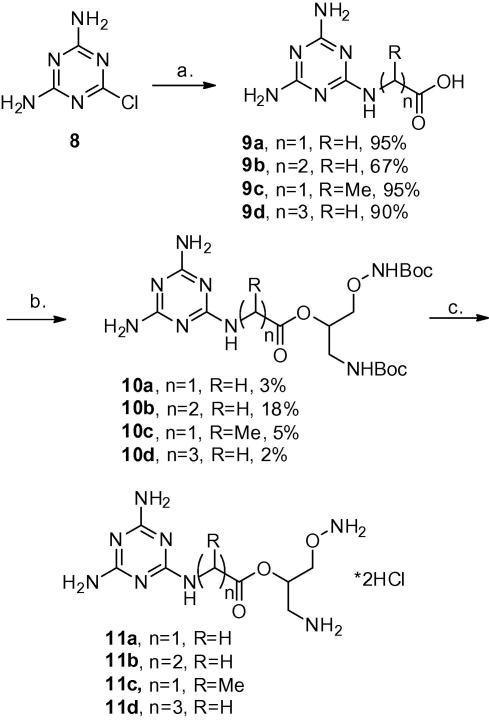
Reagents and conditions: (a) amino acid, NaHCO_3_, EtOH/water; 80 °C; (b) (i) SOCl_2_, DCM, (ii) **6**, DMAP, Et_3_N, CH_3_CN, 50 °C; (c) 2 M HCl in Et_2_O, 99%.

**Table 1:** In vitro activity of compounds against *T. brucei* ODC and *T. brucei*

Compd	*T. brucei* ODC IC_50_ (nM)	*T. brucei brucei* EC_50_ (μM)		Resistance factor^a^
		Wild type	*TbAT1* KO	
**3**	22 ± 7	>100		
**7**	48 ± 9	NA		
**11a**		>100	NA	
**10b**		77	40	0.5
**11b**		>100	NA	
**11c**		29	NA	>3
**11d**		113	288	2.5

a EC_50_ KO/EC_50_ w.t. NA—no activity at 100 μM.

## Supplementary data

Supplementary data Experimental procedures.
