# Supplementary material for: Selective delivery of 2-hydroxy APA to Trypanosoma brucei using the melamine motif
Source: Bioorg Med Chem Lett. 2010 Aug 1;20(15):4364–6. doi: 10.1016/j.bmcl.2010.06.070 (PMC2935964; doi:10.1016/j.bmcl.2010.06.070)
Supplement: Supplementary data — Experimental procedures. [file mmc1.pdf]

Selective delivery of 2-hydroxy APA to *Trypanosoma brucei* using the melamine motif

Nina Klee[a], Pui Ee Wong[b] Beatriz Baragaña[a], Farah El Mazouni,[c] Margaret A. Phillips,[c] Michael P. Barrett[b], Ian H. Gilbert.[a,*]

[a]*Division of Biological Chemistry and Drug Discovery, College of Life Science, University of Dundee, Sir James Black Centre, Dundee DD1 5EH, UK.* [b]*Division of Infection and Immunity and Wellcome Trust Centre for Molecular Parasitology, Glasgow Biomedical Research Centre, University of Glasgow G12 8TA, UK.* [c]*UT Southwestern Medical Center at Dallas 6001 Forest Park, Dallas, Texas 75390-9041, USA.*

Experimental

General information

Chemicals were purchased form Sigma-Aldrich and were used without further purification. Dry solvents were purchased form Fluka in sure-seal bottles stored over molecular sieves. Deuterated solvents were purchased form Gross.
Microwave-assisted reactions were carried out using the automated Biotage Initiatior Systems.
Purification by flash column chromatography was performed using a Combiflash Companion system and Presearch® or Silicyle® propylene columns, which were prepacked with silica.
Qualitative thin layer chromatography (TLC) was carried out on pre-coated aluminium sheets silica gel (Kieselgel 60 F_{254}, BDH). Compounds were detected with ninhydrin, $KMnO_4$ or 254nm UV light.
Melting points were determined with a Gallenkamp melting point apparatus and are not corrected.
^{1}H-NMR spectra were recorded at a Bruker 500 MHz NMR spectrometer using the applied solvent simultaneously as internal standard. Chemical shifts (δ) are given in ppm together with the multiplicity, the coupling constants (J(H,H)/Hz), relative frequency and assignment of the observed signal. Chemical shift of AB systems are directly deduced form Mestre-C free software or Bruker Topsip programme. (For NMR values given s = singlet, d=doublet, t=triplet, q=quartet, qn=quintet, m=multiplet, bs=broad singlet). ^{13}C-NMR spectra were recorded at a Bruker 500 MHz NMR spectrometer using the applied solvent simultaneously as internal standard. Chemical shifts (δ) are given in ppm.
Mass spectra were recorded at a MicroTOF mass spectrometer form Bruker Daltonics, where ionisation was achieved in the positive electrospray mode. High resolution mass spectra were also recorded at a MicroTOF mass spectrometer from Bruker Daltonics. Accurate mass was performed by peak matching.

tert-Butyl _N_-[3-(_tert_-butoxycarbonylamino)oxypropyl]carbamate (2)
tert-Butyl *N*-hydroxycarbamate (0.399 g, 3.0 mmol) was slowly added to a solution of NaH (60 % dispersion in mineral oil, 0.120 g, 3.0 mmol) in THF (10ml) at 0°C. After stirring for 30 minutes at 0°C, *tert*-butyl *N*-(3-bromopropyl)carbamate (0.357 g, 1.5 mmol) was added. The mixture was allowed to reach room temperature and continue stirring overnight. Then, isopropanol (1ml) was added and the mixture was poured into ice- water (70 ml), extracted with DCM (3 × 100 ml), dried ($MgSO_4$) and the solvent removed under reduced pressure. The reaction crude was purified by column chromatography (ethyl acetate/n-hexane : 1/5-1/3) to obtain **1** as a colourless solid (0.184 g, 42%). ^{1}H NMR (500 MHz; $CDCl_3$) δ 5.16 (m, 1H, NH), 3.94 (t, J= 5.9 Hz, 2H, O-CH_2-CH_2), 3.29 (dt, J = 6.1Hz, J = 6.2 Hz, 2H,

CH_2-CH_2-NH), 1.81 (m, 2H, CH_2-CH_2-CH_2), 1.51 (s, 9H, -C- (CH_3)$_3$), 1.46 (s, 9H, -C-(CH_3)$_3$). ^{13}C NMR (125 MHz; $CDCl_3$) δ 161.7 (C), 85.1 (C), 83.0 (C), 77.9 (CH_2), 41.1 (CH_2), 33.4 (CH_2), 31.9 (CH_3), 31.8 (CH_3). LRMS (ES^+): m/z 291.2 ($(M+H)^+$, 24%), 135 (6%), 179 (100%), 235 (19%), 603 ($2(M+H)^+$, 24%)). HRMS (ES+): m/z calcd mass for $(C_{13}H_{27}N_2O_5)^+$: 291.1914 found 291.1922.

3-(Aminooxy)propan-1-amine hydrochloride (3)
Compound **2** (0.092 g, 0.32 mmol) was dissolved in chloroform (3ml) and 2M HCl in Ether (1ml) was added. The reaction was stirred overnight until no starting material could be detected by TLC and the white precipitate was filtered and washed with DCM (1ml). After drying under reduced pressure compound **3** was obtained as a white powder (0.042 g, 82%). mp 205 °C; ^{1}H NMR (500 MHz; D_2O) δ 4.04 (t, J = 5.8 Hz, 2H, O-CH_2-CH_2), 3.00 (t, J = 7.5 Hz, 2H, NH_2-CH_2-CH_2), 1.96-1.91 (m, 2H, CH_2-CH_2-CH_2). ^{13}C NMR (125 MHz; D_2O) δ 72.3 (CH_2), 36.6 (CH_2), 25.2 (CH_2). LRMS (ES^+): m/z 91.2 ($(M+H)^+$, 62%), 76.2 (100%). HRMS (ES+): m/z calcd mass for $(C_3H_{11}N_2O)^+$: 91.0865 found 91.0866.

***tert*-Butyl *N*-(2-oxiranylmethyl)carbamate (5)**
tert-Butyl *N*-allylcarbamate (3.14 g, 20.0 mmol) was dissolved in DCM (200ml) and cooled to 0°C. MCPBA (6.88 g, 40 mmol) was added portion wise and the solution stirred at room temperature for two days. The mixture was washed with 10% $Na_2SO_{3(aq)}$ (100ml), $NaHCO_3$ (200ml) and brine (2 × 200ml). The organic layer was dried over $MgSO_4$ and the solvent removed under reduced pressure to give pure compound **5** as colourless oil. (3.43 g, 99%). ^{1}H NMR (500 MHz; $CDCl_3$) δ 4.65 (bs, 1H, NH), 3.27-3.23 (m, 1H, CH-CH_2-NH), 2.94 (ddd, J = 5.2 Hz, J = 6.2 Hz, J = 14.7 Hz, 1H, CH-CH_2-NH), 2.81 (bs, 1H, CH_2-O-CH-CH_2), 2.50 (dd, J = 4.1 Hz, J = 4.6 Hz, 1H, CH_2-O-CH), 2.32 (dd, J = 2.7 Hz, J = 4.7 Hz, 1H, CH_2-O-CH), 1.17 (s, 9H, -C-(CH_3)$_3$). ^{13}C NMR (125 MHz; $CDCl_3$) δ 155.9 (C), 79.5 (C), 50.8 (CH), 45.0 (CH_2), 41.7 (CH_2), 28.3 (CH_3). LRMS (ES^+): m/z 347.2 ($(2M+H)^+$, 16%), 174.1 ($(M+H)^+$, 100%). HRMS (ES+): m/z calcd mass for $(C_8H_{16}NO_3)^+$: 174.1125 found 174.1132.

***N,N'*-Bis[(*tert*-butyloxy)carbonyl]-1-amino-3-(aminooxy)-2-propanol (6)**

tert-Butyl *N*-(2-oxiranylmethyl)carbamate **(5)** (3.43 g, 20.0 mmol) was dissolved in ethanol (20ml) and *tert*-butyl *N*-hydroxycarbamate (2.66 g, 20.0 mmol) and triethylamine (5.53ml, 40mmol) were added at room temperature. The solution was heated at 80 °C and stirred at this temperature for 2 days. The solvent was removed under reduced pressure and the remaining oil dissolved in DCM (60ml) and washed with brine (3x60ml). Purification by column chromatography (ethyl acetate/n-hexane : 1/5 to 1/3) gave the title compound **6** as a white powder (2.4 g, 39.4%). ^{1}H NMR (500 MHz; $CDCl_3$) δ 7.36 (s, 1H, CHOH), 5.11 (bs, 1H, NH), 4.56 (bs, 1H, NH), 3.98-3.96 (m, 1H, CH_2-CHOH-CH_2), 3.87 (dd, 1H, J = 3.0 Hz, J = 11.5 Hz, CHOH-CH_2-O), 3.73 (dd, 1H, J = 8.9 Hz, J = 11.4 Hz, CHOH-CH_2-O), 3.40-3.35 (m, 1H, NH-CH_2-CHOH), 3.16-3.11 (m, 1H, NH-CH_2-CHOH), 1.50 (s, 9H, -C-(CH_3)$_3$), 1.46 17 (s, 9H, -C-(CH_3)$_3$). ^{13}C NMR (125 MHz; $CDCl_3$) δ 158.4 (C), 156.4 (C), 82.4 (C), 79.0 (C), 67.8 (CH_2), 42.3 (CH), 31.5 (CH_2), 28.3 (CH_3) 28.1 (CH_3). LRMS (ES^+): m/z 635.3 ($(2M+H)^+$, 14%), 307.2 ($(M+H)^+$, 29%), 251.1 (29%), 195.1 (100%), 151.1 (19%). HRMS (ES+): m/z calcd mass for $(C_{13}H_{26}N_2NaO_6)^+$: 329.1683 found 329.1680.

1-Amino-3-(aminoxy)-2-propanol Dihydrochloride (7)
Compound **6** (0.092 g, 0.30 mmol) was dissolved in chloroform (3ml) and 2M HCl in Ether (1ml) was added. The reaction was stirred overnight until no starting material could be detected by TLC and the white precipitate was filtered and washed with DCM (1ml). After drying under reduced pressure compound **7** was obtained as a white powder (0.043 g, 80%). mp 156 °C (lit.[14] mp 155-156°C). ^{1}H

NMR (500 MHz; D_2O) δ 4.10-4.03 (m, 2H, CH_2-C*H*OH-CH_2 and CHOH-C*H*$_2$-O), 3.97 (ddd, *J*= 2.1 Hz, *J*= 5.2 Hz, *J*= 9.9 Hz, 1H, CHOH-C*H*$_2$-O), 3.08 (dd, *J*= 3.0 Hz, *J*= 13.3 Hz, 1H, NH_2-C*H*$_2$-CHOH), 2.95 (dd, *J*= 9.5 Hz, *J*= 13.3 Hz, 1H, NH_2-C*H*$_2$-CHOH), ^{13}C NMR (125 MHz; D_2O) δ 75.7 (CH_2), 65.3 (CH), 41.0 (CH_2). LRMS (ES^+): m/z 107.3 ($(M+H)^+$, 59%); 92.2 (100%). HRMS (ES+): m/z calcd mass for $(C_3H_{11}N_2O_2)^+$: 107.0815 found 107.0817.

2-(4,6-diamino-1,3,5-triazin-2-ylamino)ethanoic acid (9a)

2-Chloro-4,6-diamino-1,3,5-triazine (1.45 g, 10.0 mmol), glycine (1.13 g, 15 mmol) and $NaHCO_3$ (1.68g, 20mmol) were suspended in Ethanol/Water 1/1 (225 ml). The suspension was stirred at 80 °C for 2 days, becoming a solution. After cooling to room temperature, the product was precipitated by adjusting the pH to 3-4 with 0.5 M HCl. The white precipitate was filtered and washed with cold water (1ml) to give compound **9a** (1.41 g, 95%). ^{1}H NMR (500 MHz; DMSO-d_6) δ 6.63 (t, *J*= 6.3 Hz, 1H, N*H*), 6.23 (m, 2H, N*H*$_2$), 6.14 (m, 2H, N*H*$_2$), 3.84 (d, *J*= 6.3 Hz, 2H, NH-C*H*$_2$-CO). ^{13}C NMR (125 MHz; DMSO-d_6) δ 172.4 (C), 166.8 (C), 166.6 (C), 166.2 (C), 41.9 (CH_2). LRMS (ES^+): m/z 369.1 ($(2M+H)^+$, 4%), 185.1 ($(M+H)^+$, 100%). HRMS (ES+): m/z calcd mass for $(C_5H_9N_6O_2)^+$: 185.0781 found 185.0790.

3-(4,6-diamino-1,3,5-triazin-2-ylamino)propanoic acid (9b)

2-Chloro-4,6-diamino-1,3,5-triazine (1.45 g, 10.0 mmol), β-alanine (1.34 g, 15 mmol) and $NaHCO_3$ (1.68g, 20mmol) were suspended in Ethanol/Water 1/1 (225 ml). The suspension was stirred at 80 °C for 2 days, becoming a solution. After cooling to room temperature, the product was precipitated by adjusting the pH to 3-4 with 0.5 M HCl. The white precipitate was filtered and washed with cold water (1ml) to give compound **9b** (1.32 g, 67%). ^{1}H NMR (500 MHz; DMSO-d_6) δ 6.49 (t, *J* = 5.7 Hz, 1H, N*H*), 6.24-6.11 (m, 4H, N*H*$_2$), 3.38 (dt, *J* = 6.2 Hz, *J* = 6.8 Hz, 2H, NH-C*H*$_2$-CH_2), 2.45 (t, *J* = 7.1Hz, 2H, CH_2-C*H*$_2$-CO). ^{13}C NMR (125 MHz; DMSO-d_6) δ 173.5 (C), 167.0 (C), 166.4 (C), 165.8 (C), 36.0 (CH_2), 30.7 (CH_2). LRMS (ES^+): m/z 397.2 ($(2M+H)^+$, 6%), 199.1 ($(M+H)^+$, 100%). HRMS (ES+): m/z calcd mass for $(C_6H_{11}N_6O_2)^+$: 199.0938 found 199.0933.

2-(4,6-diamino-1,3,5-triazin-2-ylamino)propanoic acid (9c)

2-Chloro-4,6-diamino-1,3,5-triazine (1.45 g, 10.0 mmol), D,L- alanine (1.34 g, 15 mmol) and $NaHCO_3$ (1.68g, 20mmol) were suspended in Ethanol/Water 1/1 (225 ml). The suspension was stirred at 150 °C for 4h in the microwave. After cooling to room temperature, 0.5 M HCl was added to adjust the pH to 3-4. The solvent was removed under reduced pressure and the remaining solid triturated with cold water (2ml), filtered and washed with cold water (1ml) to give compound **9c** (1.87 g, 95%). ^{1}H NMR (500 MHz; DMSO-d_6) δ 8.46 (bs, 1H, N*H*), 8.04-7.94 (m, 4H, N*H*$_2$), 4.44 (qn, *J*= 7.3 Hz, 1H, CH_3-C*H*-NH), 1.36 (d, *J*= 7.3 Hz, 3H, CH-C*H*$_3$); ^{13}C NMR (125 MHz; DMSO-d_6) δ 173.4 (C), 158.4 (C), 156.2 (C), 48.9 (CH), 17.0 (CH_3); LRMS (ES^+): m/z 397.2 ($(2M+H)^+$, 5%), 199.1 ($(M+H)^+$, 100%). HRMS (ES+): m/z calcd mass for $(C_6H_{11}N_6O_2)^+$: 199.0938 found 199.0938.

4-(4,6-diamino-1,3,5-triazin-2-ylamino)butanoic acid (9d)

2-Chloro-4,6-diamino-1,3,5-triazine (1.45 g, 10.0 mmol), γ-aminobutanoic acid (1.43 g, 15 mmol) and $NaHCO_3$ (1.68g, 20mmol) were suspended in Ethanol/Water 1/1 (225 ml). The suspension was stirred at 80 °C for 2 days, becoming a solution. After cooling to room temperature, 0.5 M HCl was added to adjust the pH to 3-4. The solvent was removed under reduced pressure and the remaining solid triturated with cold water (2ml), filtered and washed with cold water (1ml) to give compound **9d** (1.91 g, 90%). ^{1}H NMR (500 MHz; DMSO-d_6) δ 8.27 (bs, 1H, N*H*), 8.09-7.86 (m, 4H, N*H*$_2$), 3.34-3.24 (m,

2H, NH-CH_2-CH_2), 2.27 (t, J = 7.4 Hz, 2H, CH2-CH_2-CO), 1.77-1.68 (m, 2H, CH_2-CH_2-CH_2). ^{13}C NMR (125 MHz; DMSO-d_6) δ 174.0 (C), 156.0 (C), 155.9 (C), 30.9 (CH_2), 24.1 (CH_2). LRMS (ES^+): m/z 425.2 ($(2M+H)^+$, 4%), 213.1 ($(M+H)^+$, 100%). HRMS (ES+): m/z calcd mass for $(C_7H_{13}N_6O_2)^+$: 213.1095 found 213.1103.

_N,N'_-Bis[(_tert_-butyloxy)carbonyl]-1-amino-3-(aminooxy)-2-propyl 2-(4,6-diamino-1,3,5-triazin-2-ylamino)acetate (10a)

Compound **9a** (0.588 g, 3.2 mmol) was suspended in thionylchloride (6 ml) and DCM (6ml). The suspension was stirred at room temperature under argon for 24 hours. Toluene was added (2ml) and the solvents removed under reduced pressure. Then, the resulting yellowish powder, compound **6** (0.490 g, 1.6 mmol) and DMAP (0.394 g, 3.2mmol) were dissolved in acetonitrile (10ml) under argon. Triethylamine (0.44ml, 3.2 mmol) was added and the red suspension was stirred at 50 °C for 2 days. The solvent was removed under reduced pressure and the crude mixture was purified by column chromatography (ethyl acetate/n-hexane: 3/10) to recover un-reacted **6** (406 mg) and then (methanol/DCM: 1/10) to obtain pure **10a** as an amorphous solid. The solid was dissolved in methanol (1ml), water was added (30 ml) and the suspension frozen immediately. Freezdrying gave **10a** as a white powder (22 mg, 2.9%). ^{1}H NMR (500 MHz; MeOD-d_4) δ 5.07-5.04 (m, 1H, CH_2-CHOH-CH_2), 3.98 (d, J = 1.9 Hz, 2H, NH-CH_2-CO), 3.83 (dd, J = 4.1 Hz, J = 11.4 Hz, 1H, CH-CH_2-O), 3.77 (dd, J = 6.4 Hz, J = 11.3 Hz, 1H, CH-CH_2-O), 3.32-3.28 (m, 1H, NH-CH_2-CH), 3.16-3.11 (m, 1H, NH-CH_2-CH), 1.37 (s, 9H, -C-(CH_3)$_3$), 1.33 (s, 9H, -C-(CH_3)$_3$). ^{13}C NMR (125 MHz; MeOD-d_4) δ 172.0 (C), 168.3 (C), 167.7 (C), 159.1 (C), 158.5 (C), 82.4 (C), 80.4 (C), 76.2 (CH), 72.6 (CH_2), 43.6 (CH_2), 41.5 (CH_2), 28.8 (CH_3), 28.6 (CH_3). LRMS (ES^+): m/z 473.3 ($(M+H)^+$, 100%). HRMS (ES+): m/z calcd mass for $(C_{18}H_{33}N_8O_7)^+$: 473.2467 found 473.2453.

_N,N'_-Bis[(_tert_-butyloxy)carbonyl]-1-amino-3-(aminooxy)-2-propyl 3-(4,6-diamino-1,3,5-triazin-2-ylamino)propanoate (10b)

Compound **9b** (0.158 g, 0.8 mmol) was suspended in thionylchloride (6 ml) and DCM (6ml). The suspension was stirred at room temperature under argon for 24 hours. Toluene was added (2ml) and the solvents removed under reduced pressure. Then, the resulting white powder, compound **6** (0.490 g, 1.6 mmol) and DMAP (0.098 g, 0.8 mmol) were dissolved in acetonitrile (10ml) under argon. Triethylamine (0.11ml, 0.8 mmol) was added and the white suspension was stirred at 50 °C for 2 days. The solvent was removed under reduced pressure and the crude mixture purified by column chromatography (ethyl acetate/n-hexane: 3/10) to recover un-reacted **6** (290 mg) and then (methanol/DCM : 1/10) to obtain pure **10b** as an amorphous solid. The solid was dissolved in methanol (1ml), water was added (30 ml) and the suspension frozen immediately. Freezdrying gave **10b** as a white powder (70 mg, 18.0%). ^{1}H NMR (500 MHz; MeOD-d_4) δ 5.14 (bs, 1H, CH_2-CHOH-CH_2), 3.95-3.86 (m, 2H, CH-CH_2-O), 3.61 (bs, 2H, NH-CH_2-CH_2), 3.42-3.22 (m, 2H, NH-CH_2-CH), 2.65-2.62 (m, 2H, CH_2-CH_2-CO), 1.49 (s, 9H, -C-(CH_3)$_3$), 1.45 (s, 9H, -C-(CH_3)$_3$). ^{13}C NMR (125 MHz; MeOD-d_4) δ 173.3 (C), 167.4 (C), 159.2 (C), 158.5 (C), 82.4 (C), 80.3 (C), 76.3 (CH), 72.0 (CH_2), 41.4 (CH_2), 37.3 (CH_2), 35.5 (CH_2), 28.8 (CH_3), 28.6 (CH_3); LRMS (ES^+): m/z 487.3 ($(M+H)^+$, 100%). HRMS (ES+): m/z calcd mass for $(C_{19}H_{35}N_8O_7)^+$: 487.2623 found 487.2626.

N,N'-Bis[(*tert*-butyloxy)carbonyl]-1-amino-3-(aminooxy)-2-propyl 2-(4,6-diamino-1,3,5-triazin-2-ylamino)propanoate (10c)

Compound **9c** (0.376 g, 1.9 mmol) was suspended in thionylchloride (6 ml) and DCM (6ml). The suspension was stirred at room temperature under argon for 24 hours. Toluene was added (2ml) and the solvents removed under reduced pressure. Then, the resulting white powder, compound **6** (0.612 g, 2 mmol) and DMAP (0.232 g, 1.9 mmol) were dissolved in acetonitrile (10ml) under argon. Triethylamine (0.26ml, 1.9 mmol) was added and the white suspension was stirred at 50 °C for 2 days. The solvent was removed under reduced pressure and the crude mixture purified by column chromatography (ethyl acetate/n-hexane: 3/10) to recover un-reacted **6** (304 mg) and then (Methanol/DCM: 1/10) to obtain **10c.** Compound **10c** was further purified by reverse phase chromatography (methanol/water: 1/1) and then suspended in methanol (1ml) and water (30 ml) and frozen immediately. Freezdrying gave **10c** as a white powder (51 mg, 5.5%). ^{1}H NMR (500 MHz; MeOD-d_4) δ 5.19-5.14 (m, CH_2-C*H*OH-CH_2), 4.51-4.54 (m, 1H, NHC*H*CH_3), 3.99-3.87 (m, 2H, CH-CH_2-O), 3.44-3.26 (m, 2H, NH-CH_2-CHOH), 1.48-1.43 (m, 21H, CH_3-CH and -C-(CH_3)$_3$). ^{13}C NMR (125 MHz; MeOD-d_4) δ 175.1 (C), 168.9 (C), 168.4 (C), 159.1 (C), 158.5 (C), 82.5 (C), 80.5 (C), 76.3 (CH), 72.6 (CH_2), 50.9 (CH) ,41.7 (CH_2), 28.6 (CH_3), 28.8 (CH_3), 18.1 (CH_3). LRMS (ES$^+$): m/z 487.3 ((M+H)$^+$, 100%). HRMS (ES+): m/z calcd mass for $(C_{19}H_{35}N_8O_7)^+$: 487.2623 found 487.2603.

N,N'-Bis[(*tert*-butyloxy)carbonyl]-1-amino-3-(aminooxy)-2-propyl 4-(4,6-diamino-1,3,5-triazin-2-ylamino)butanoate (10d)

Compound **9d** (0.191 g, 0.9 mmol) was suspended in thionylchloride (6 ml) and DCM (6ml). The suspension was stirred at room temperature under argon for 24 hours. Toluene was added (2ml) and the solvents removed under reduced pressure. The resulting white powder, compound **6** (0.612 g, 2 mmol) and DMAP (0.110 g, 0.9 mmol) were dissolved in acetonitrile (10ml) under argon. Triethylamine (0.12ml, 0.9 mmol) was added and the white suspension was stirred at 50 °C for 2 days. The solvent was removed under reduced pressure and the crude mixture purified by column chromatography (ethyl acetate/n-hexane: 3/10) to recover un-reacted **6** (344 mg) and then (methanol/DCM: 1/10) to obtain **10d.** Compound **10d** was further purified by reverse phase chromatography (methanol/water: 1/1) and then suspended in methanol (1ml) and water (30 ml) and frozen immediately. Freeze drying gave **10d** as a white powder (6 mg, 1.6%). ^{1}H NMR (500 MHz; MeOD-d_4) δ 5.14- 1.12 (m, 1H, CH_2-C*H*OH-CH_2), 3.95-3.87 (m, 2H, CH-CH_2-O), 3.43-3.35 (m, 3H, NH-CH_2-CHOH and NH-CH_2-CH_2), 3.28-3.22 (m, 1H, NH-CH_2-CHOH), 2.44 (dt, J = 1.8Hz, J = 7.2 Hz, 2H, CH_2-CH_2-CO), 1.91 (m, 2H, CH_2-CH_2-CH2), 1.48 (s, 9H, -C-(CH_3)$_3$), 1.44 (s, 9H, -C-(CH_3)$_3$). ^{13}C NMR (125 MHz; MeOD-d_4) δ 174.9 (C), 167.2 (C), 159.2 (C), 158.6 (C), 82.6 (C), 80.5 (C), 76.3 (CH), 71.9 (CH_2), 41.4 (CH_2), 40.8 (CH_2), 32.7 (CH_2), 28.8 (CH_3), 28.6 (CH_3), 26.0 (CH_2). LRMS (ES$^+$): m/z 501.3 ((M+H)$^+$, 100%). HRMS (ES+): m/z calcd mass for $(C_{20}H_{37}N_8O_7)^+$: 501.2780 found 501.2771.

1-Amino-3-(aminoxy)-2-propyl 2-(4,6-diamino-1,3,5-triazin-2-ylamino)acetate Dihydrochloride (11a)

Compound **10a** (0.058 g, 0.12 mmol) was dissolved in DCM (2ml) and HCl in ethyl ether (2M, 0.3ml) was added slowly. The solution was stirred under argon for one hour. The solvent was removed and fresh DCM (2ml) and HCl in ethyl ether (2M, 0.3ml) were added. The reaction was stirred for further 3 hours under argon. The suspension was allowed to settle and most of the solvent was decanted with a pipette. Drying under reduced pressure gave **11a** as a white powder (42 mg, 100%). mp 150-170. ^{1}H NMR (500 MHz; MeOD-d_4) δ 5.35-5.30 (m, 1H, CH_2-C*H*OH-CH_2), 4.30-4.21 (m, 4H, CHOH-CH_2-O and NH-CH_2-CO), 3.31-3.23 (m, 2H, CHOH-CH_2-NH_2). ^{13}C NMR (125 MHz; MeOD-d_4) δ 170.6

(C), 163.4 (C), 161.1 (C), 158.4 (C), 74.5 (CH), 70.1 (CH_2), 43.5 (CH_2), 40.7 (CH_2). LRMS (ES^+): m/z 137.1 ($(M+2H)^{2+}$, 15%), 185.1 (100%), 255.1 (65%), 273.1 ($(M+H)^+$, 45%), 545.3 ($(2M+H)^+$, 8%). HRMS (ES+): m/z calcd mass for $(C_8H_{17}N_8O_3)^+$: 273.1418 found 273.1422.

1-Amino-3-(aminoxy)-2-propyl 3-(4,6-diamino-1,3,5-triazin-2-ylamino)propanoate Dihydrochloride (11b)

Compound **10b** (0.126 g, 0.26 mmol) was dissolved in DCM (4ml), HCl in ethyl ether (2M, 0.6ml) was added slowly and the solution was stirred under argon for one hour. The solvent was removed and fresh DCM (4ml) and HCl in ethyl ether (2M, 0.6ml) were added. The reaction was stirred for further 3 hours under argon. The suspension was allowed to settle and most of the solvent was decanted with a pipette. Drying under reduced pressure gave **11b** as a white powder (93 mg, 100%). mp 150-170. ^1H NMR (500 MHz; MeOD-d_4) δ 5.30-5.26 (m, 1H, CH_2-C*H*OH-CH_2), 4.27-4.20 (m, 2H, CHOH-CH_2-O), 3.61 (t, J = 6.6Hz, 2H, NH-CH_2-CH_2), 3.28-3.18 (m, 2H, CHOH-CH_2-NH_2), 2.78-2.66 (m, 2H, CH_2-CH_2-CO). ^{13}C NMR (125 MHz; MeOD-d_4) δ 172.2 (C), 162.1 (C), 161.6 (C), 158.1 (C), 74.7 (CH), 69.3 (CH_2), 40.8 (CH_2), 37.5 (CH_2), 34.5 (CH_2). LRMS (ES^+): m/z 144.1 ($(M+2H)^{2+}$, 20%), 199.1 (100%), 269.1 (20%), 287.2 ($(M+H)^+$, 75%), 573.3 ($(2M+H)^+$, 5%). HRMS (ES+): m/z calcd mass for $(C_9H_{19}N_8O_3)^+$: 287.1575 found 287.1578.

1-Amino-3-(aminoxy)-2-propyl 2-(4,6-diamino-1,3,5-triazin-2-ylamino)propanoate Dihydrochloride (11c)

Compound **10c** (0.046 g, 0.095 mmol) was dissolved in DCM (2ml), HCl in ethyl ether (2M, 0.2ml) was added slowly and the solution was stirred under argon for one hour. The solvent was removed and fresh DCM (2ml) and HCl in ethyl ether (2M, 0.2ml) were added. The reaction was stirred for further 3 hours under argon. The suspension was allowed to settle and most of the solvent was decanted with a pipette. Drying under reduced pressure gave **11c** as a white powder (34 mg, 100%). mp 160-180. ^1H NMR (500 MHz; MeOD-d_4) δ 5.45-5.38 (m, 1H, CH_2-C*H*OH-CH_2), 4.39-4.30 (m, 2H, CHOH-CH_2-O), 3.43-3.35 (m, 2H, NH_2-CH_2-CHOH), 1.57, (d, J= 8.2Hz, 3H, CH-CH_3). ^{13}C NMR (125 MHz; MeOD-d_4) δ 173.4 (C), 74.4 (CH), 70.1 (CH_2), 51.1 (CH), 40.7 (CH_2), 17.8 (CH_3), 17.5 (CH_3). LRMS (ES^+): m/z 144.1 ($(M+2H)^{2+}$, 23%), 199.1 (100%), 269.1 (40%), 287.2 ($(M+H)^+$, 55%), 573.3 ($(2M+H)^+$, 8%). HRMS (ES+): m/z calcd mass for $(C_9H_{19}N_8O_3)^+$: 287.1575 found 287.1577.

1-Amino-3-(aminoxy)-2-propyl 4-(4,6-diamino-1,3,5-triazin-2-ylamino)butanoate Dihydrochloride (11d)

Compound **10d** (0.009 g, 0.02 mmol) was dissolved in DCM (2ml), HCl in ethyl ether (2M, 0.5ml) was added slowly and the solution was stirred under argon for one hour. The solvent was removed and fresh DCM (2ml) and HCl in ethyl ether (2M, 0.2ml) were added. The reaction was stirred for further 3 hours under argon. The suspension was allowed to settle and most of the solvent was decanted with a pipette. Drying under reduced pressure gave **11d** as a white powder (7 mg, 100%). ^1H NMR (500 MHz; MeOD-d_4) δ 5.24-5.22 (m, 1H, CH_2-C*H*OH-CH_2), 3.96-3.95 (m, 2H, CHOH-CH_2-O), 3.36 (t, 2H, J=6.8Hz , NH-CH_2-CH_2), 3.21-3.12 (m, 2H, NH_2-CH_2-CHOH), 2.49-2.38 (m, CH_2-CH_2-CO), 1.86-1.80 (m, 2H, CH_2-CH_2-CH_2). ^{13}C NMR (125 MHz; MeOD-d_4) δ 174.2 (C), 75.0 (CH), 69.5 (CH_2), 41.0 (CH_2), 32.0 (CH_2), 25.3 (CH_2). LRMS (ES^+): m/z 151.1 ($(M+2H)^{2+}$, 10%), 213.1 (100%), 301.1 (60%).
